# The green peach aphid *Myzus persicae* perform better on pre-infested Chinese cabbage *Brassica pekinensis* by enhancing host plant nutritional quality

**DOI:** 10.1038/srep21954

**Published:** 2016-02-24

**Authors:** He-He Cao, Hui-Ru Liu, Zhan-Feng Zhang, Tong-Xian Liu

**Affiliations:** 1 State Key Laboratory for Crop Stress Biology in Arid Areas, Yangling, Shaanxi, 712100, China; 2 Key Laboratory of Crop Pest Management on the Northwest Loess Plateau, Ministry of Agriculture, Yangling, Shaanxi, 712100, China; 3 Innovation Experimental College, Northwest A&F University, Yangling, Shaanxi, 712100, China

## Abstract

The green peach aphid, *Myzus persicae* Sulzer, is a notorious pest on vegetables, which often aggregates in high densities on crop leaves. In this study, we investigated whether *M. persicae* could suppress the resistance level of Chinese cabbage *Brassica pekinensis*. *M. persicae* performed better in terms of weight gain (~33% increase) and population growth (~110% increase) when feeding on previously infested (pre-infested) Chinese cabbage compared with those on non-infested plants. However, when given a choice, 64% of the aphids preferred to settle on non-infested leaves, while 29% of aphids chose pre-infested leaves that had a 2.9 times higher concentration of glucosinolates. Aphid feeding significantly enhanced the amino acid:sugar ratio of phloem sap and the absolute amino acid concentration in plant leaves. Aphid infestation significantly increased the expression levels of salicylic acid (SA) marker genes, while it had marginal effects on the expression of jasmonate marker genes. Exogenously applied SA or methyl jasmonate had no significant effects on *M*. *persicae* performance, although these chemicals increased glucosinolates concentration in plant leaves. *M*. *persicae* infestation increase amino acid:sugar ratio and activate plant defenses, but aphid performed better on pre-infested plants, suggesting that both nutrition and toxics should be considered in insect-plant interaction.

Plants can limit insect herbivore performance by producing toxic metabolites or by reducing their nutritional value to insects^1^. Jasmonates play a major role in plant defence against insects by regulating defence compounds in plants that have antixenosis or antibiosis effects on insects^2^. Some plant proteins, like polyphenol oxidases and trypsin inhibitors, can reduce the nutritional value of plants for insects^2^. Insects, however, have evolved strategies to adapt to or manipulate plant defences^3^. Some insects can enhance host plant nutrition or suppress effective jasmonates signaling defences and thus perform better on pre-infested plants^3^,^4^,^5^.

Jasmonates play a critical role in regulating direct and indirect defences of plants against insects^2^. Jasmonates induce the synthesis of a range of plant defence metabolites, including nicotine, glucosinolates, flavonoids, green leaf volatiles, and terpenoid indole alkaloids^6^,^7^. Exogenously applied jasmonates reduce insect preference and performance^2^,^8^,^9^. When the jasmonate synthesis process was silenced, the native tobacco, *Nicotiana attenuata*, became more susceptible to insect herbivores and attracted novel pests that do not naturally feed on tobacco^10^.

Glucosinolates are present in all Brassicaceae, and have long been known for their role in plant defence and can be induced by jasmonates^6^,^11^. Plant release the myrosinases during tissue damage, and these endogenous enzymes hydrolyze glucosinolates to metabolites that are deterrentto generalist herbivores^12^. But some specialist insects, like *Plutella xylostella* (L.) and *Pieris rapae* (L.), rely on glucosinolates for host plant selection^12^. Some studies shown that aphid infestation enhances the concentration of glucosinolates and glucosinolates confer resistance to aphids, whereas other research found no positive or negative correlation between glucosinolate concentration and aphid performance^13^,^14^,^15^,^16^,^17^,^18^,^19^.

As phloem-feeding insects, aphids use their special mouthparts, the stylets, to obtain their nutrition from plant phloem sap. The amino acids in plant phloem are the main nutrition for phloem-feeding insects^20^. When feeding on a plant, aphids insert their stylets into the plant epidermis, regularly puncture plant mesophyll cells, and ingest cytosolic contents to decide to feed or leave the leaf well before contacting phloem sap^21^. Thus, feeding attractants or deterrents in plant mesophyll cells have strong impacts on aphid host choice^21^.

Aphid infestation usually accelerates leaf senescence and thus changes the nutritional quality of host plant for aphids^19^,^22^. Leaf senescence is the final stage of leaf development that involves degradation of macromolecules and subsequent relocation of nutrients to other parts of the plant^22^. The degradation of protein leads to increase of free amino acid in senescing leaves and their phloem sap^23^. In this case, leaf senescence may benefit the phloem-feeding insects.

The green peach aphid, *Myzus persicae* Sulzer, is a major pest of crops and vegetables worldwide. Feeding by *M. persicae* removes photoassimilates and transmits viruses, causing substantial losses of crop yield. However, this aphid species usually feeds aggregately rather than separately on plant leaves. Feeding together, however, may lead to nutrition competition, and cause more severe plant defence responses. We hypothesized that *M*. *persicae* may have evolved strategies to overcome the drawbacks of feeding aggregately. The Chinese cabbage, *Brassica pekinensis*, which is an important vegetable in China, Korea, and Japan, is often attacked by *M*. *persicae*. In this study we evaluated the preference and performance of *M*. *persicae* on non-infested and (previously infested) pre-infested Chinese cabbage. Then, we examined the amino acid and glucosinolate concentration in control and pre-infested plant leaves. The jasmonic acid and SA signalling pathway gene markers in the plant were monitored by real-time quantitative PCR. In addition, we tested the role of jasmonate and SA signalling on Chinese cabbage resistance against *M*. *persicae* by exogenous application of methyl jasmonate (MeJA) and SA.

## Results

### Table

#### *M. persicae* performance on Chinese cabbage plants

**Figure 1:**
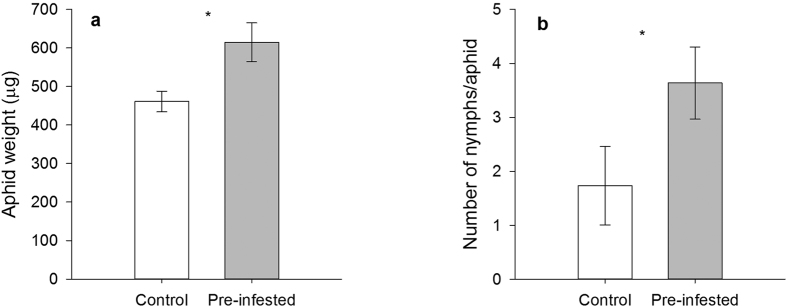
The performance of *M. persicae* on pre-infested plants and control plants. (**a**) Aphid weight after feeding for five days on non-infested plants (Control) and previously infested plants (Pre-infested). (**b**) Number of nymphs produced by per aphid after feeding for seven days. Aphids were born on treated Chinese cabbage plants and their weight or nymphs produced were examined after indicated time. Values shown are mean ± SE. *P < 0.05; Student’s *t*-test (Fig. 1a) or Mann-Whitney *U* test (Fig. 1b).

We examined the *M*. *persicae* performance on Chinese cabbage plants by measuring the weight of the aphids and this experiment repeated for four times with different sets of aphids and plants. *M. persicae* were heavier when fed on pre-infested plants for three days (*t* = 9.659, df = 16, *P* < 0.001) (see Supplementary Fig. S1a online), four days (*t* = 5.473, df = 10, *P* < 0.001) (see Supplementary Fig. S1b online), five days (*t* = 2.695, df = 14, *P* = 0.017) (Fig. 1a), and seven days (*t* = 3.7, df = 20, *P* = 0.001) (see Supplementary Fig. S1c online). Jasmonate and salicylate are two major signals of plant defence against pathogens and insects. However, neither MeJA nor SA application had significant impacts on aphid weight (ANOVA; *F*_2, 21_ = 1.525, *P* = 0.241) (data not shown). Aphids feeding on pre-infested plants produced more offspring per aphid after seven days compared with those feeding on non-infested control plants (Mann-Whitney *U* test; *P* = 0.037) (Fig. 1b).

#### *M. persicae* preferred to settle on non-infested control leaves compared with pre-infested leaves

**Figure 2:**
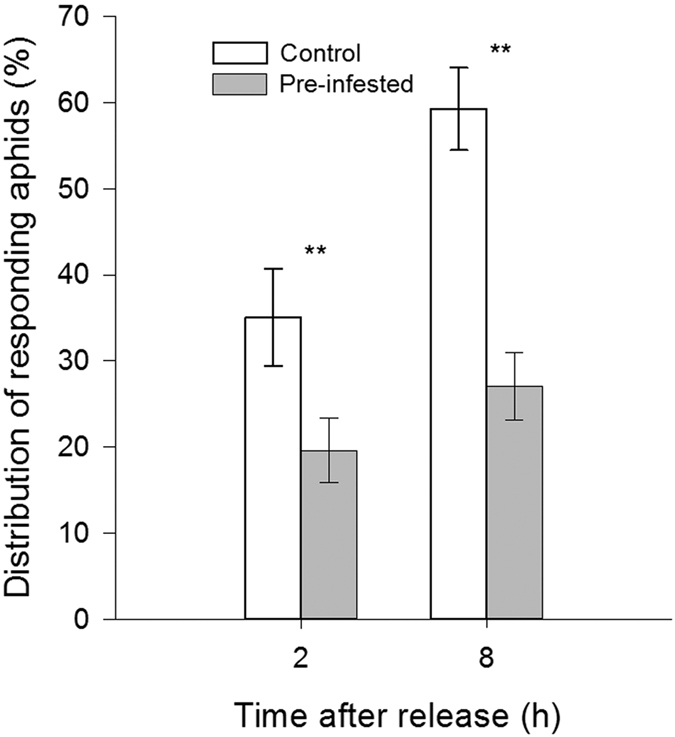
Settling preference of *M*. *persicae* on Chinese cabbage leaves. Proportion of responding *M*. *persicae* adults settled on non-infested plants (Control) and previously infested plants (Pre-infested). **P < 0.001; chi-square test. Values shown are mean ± SE.

In the choice assay, the proportion of responding apterous *M*. *persicae* adults settled on control plants were significantly higher than those on pre-infested plants at 2 h (χ^2^ = 133.327, df = 10, *P* < 0.001) and 8 h (χ^2^ = 68.962, df = 10, *P* < 0.001) (Fig. 2) after aphid release.

#### *M. persicae* infestation on Chinese cabbage plants reduced leaf chlorophyll concentration and enhanced SA marker genes expression

**Figure 3:**
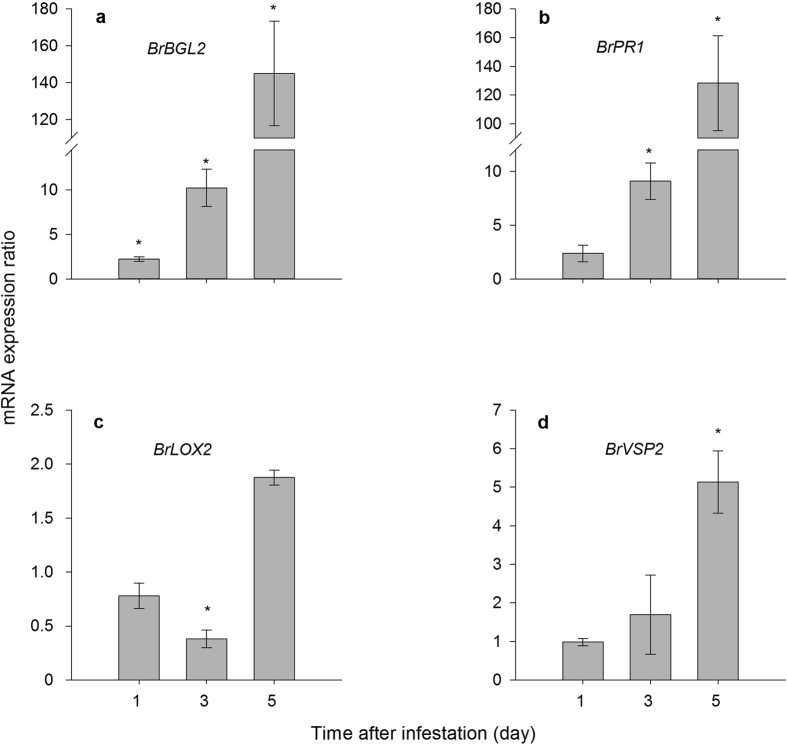
Relative expression levels of salicylic acid (**a,b**) and jasmonate (**c,d**) marker genes in *M*. *persicae* infested plants (Infested) or non-infested plants (Control). Leaves were collected and analysed after aphid feeding for one, three, and five days. The gene expression ratio of Chinese cabbage leaves infested by *M. persicae* was calculated relative to the control group, using actin gene expression for normalization. Values shown are mean ± SE. *P < 0.05. Gene abbreviations: *β-1*, *3-GLUCANASE 2* (*BrBGL2*), *PATHOGENESIS-RELATED 1* (*BrPR1*), *LIPOXYGENASE 2* (*BrLOX2*), *VEGETATIVE STORAGE PROTEIN 2* (*BrVSP2*).

**Figure 4:**
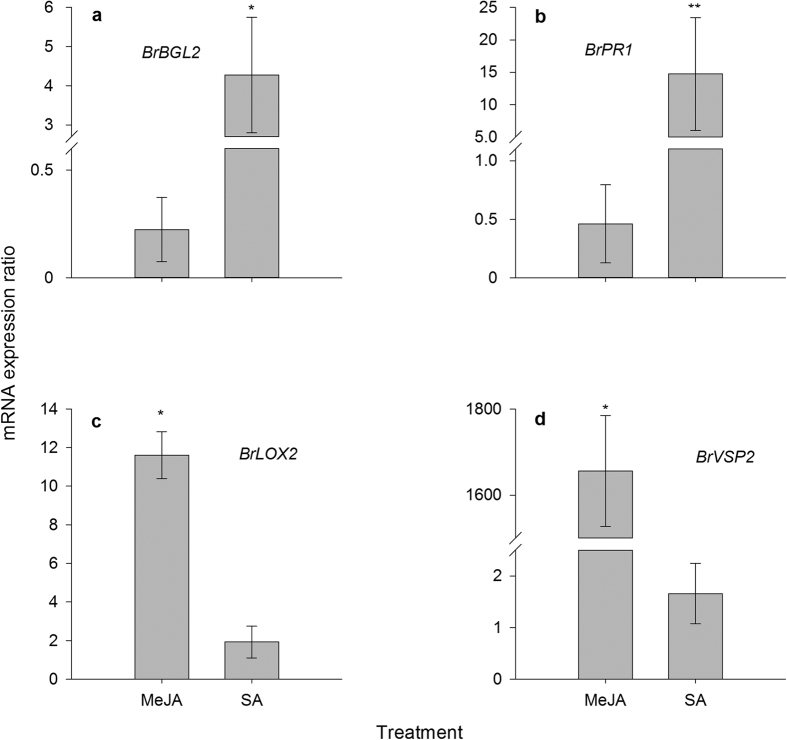
Relative expression levels of salicylic acid (SA) (**a,b**) and jasmonate (**c,d**) marker genes in SA and methyl jasmonate (MeJA) treated leaves. Leaves were collected two days after treatment. The gene expression ratio of Chinese cabbage leaves treated by MeJA or SA was calculated relative to the control group, using actin gene expression for normalization. Values shown are mean ± SE. *P < 0.05; **P < 0.01. Gene abbreviations: *β-1*, *3-GLUCANASE 2* (*BrBGL2*), *PATHOGENESIS-RELATED 1* (*BrPR1*), *LIPOXYGENASE 2* (*BrLOX2*), *VEGETATIVE STORAGE PROTEIN 2* (*BrVSP2*).

Chlorophyll concentration is an indicator of plant senescence^22^. Aphid feeding significantly reduced chlorophyll concentration (decrease 47.6%) in the Chinese cabbage leaves (*t* = 3.259, df = 14, *P* = 0.006) (see Supplementary Fig. S2 online). *M. persicae* infestation significantly increased the expression levels of SA marker genes *BrBGL2* (Fig. 3a) and *BrPR1* (Fig. 3b). The expression level of *BrBGL2* significantly increased even after one day of aphid feeding (2.3-fold increase, *P* < 0.05). Aphid feeding reduced expression level of JA marker gene *BrLOX2* after three days of feeding (decrease 61.9%, Fig. 3c), however, the expression level of JA marker gene *BrVSP2* was significantly enhanced after five days (5.1-fold increase, Fig. 3d). In addition, SA application on Chinese cabbage plants significantly increased expression levels of SA marker genes (Fig. 4a,b), while it had no influence on the expression level of JA marker genes (Fig. 4c,d). Similarly, MeJA application also significantly enhanced expression levels of JA marker genes (Fig. 4c,d), while it had no influence on the expression level of SA marker genes (Fig. 4a,b).

#### *M. persicae* feeding increased amino acid and sugar concentration in Chinese cabbage leaves

**Figure 5:**
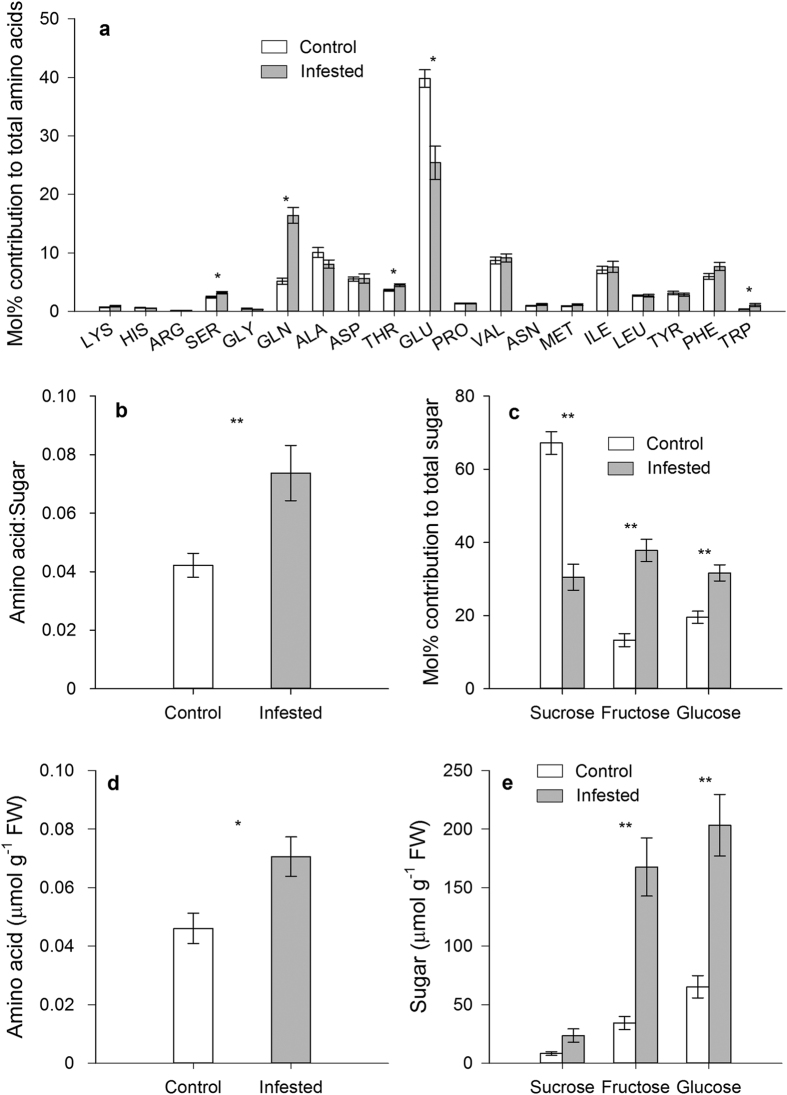
*M. persicae* feeding changed the nutritional quality of its host plants. (**a**) Relative amino acid concentration in phloem sap of non-infested plants (Control) and *M. persicae* infested plants (Infested). (**b**) The amino acid:sugar (sucrose, fructose, and glucose) ratio in phloem sap of control and aphid infested plants. (**c**) Relative sugar concentration in phloem sap of control and aphid infested plants. (**d**) Amino acid concentration and (**e**) sugar concentration in non-infested control and aphid infested (Infested) plant leaves. Values shown are mean ± SE. *P < 0.05, **P < 0.01; Student’s *t*-test.

The relative concentrations of the essential amino acids threonine (*t* = 3.163, df = 12, *P* = 0.008) and tryptophan (*t* = 3.011, df = 5, *P* = 0.047) were significantly higher in the phloem sap from infested plants (Fig. 5a). Aphid feeding also increased amino acid:sugar ratio in the phloem sap of infested plants (*t* = 3.366, df = 12, *P* = 0.006) (Fig. 5b). Total amino acid concentration in Chinese cabbage leaves was also enhanced by aphid feeding (*t* = 2.901, df = 14, *P* = 0.012) (Fig. 5d). Aphid infestation reduced relative sucrose concentration in plant phloem sap (*t* = 7.777, df = 12, *P* < 0.001) (Fig. 5c), but enhanced total sucrose concentration in plant leaves (*t* = 2.6, df = 8, *P* = 0.032) (Fig. 5e). The relative concentrations of fructose (*t* = 7.415, df = 12, *P* < 0.001) and glucose (*t* = 4.479, df = 12, *P* = 0.001) in the phloem sap of pre-infested plants were higher than in the control group (Fig. 5c). Also, aphid feeding enhanced the absolute concentrations of fructose (*t* = 5.246, df = 8, *P* = 0.001) and glucose (*t* = 4.93, df = 14, *P* < 0.001) in plant leaves (Fig. 5e).

#### Glucosinolate concentration was enhanced by aphid feeding and SA treatment

**Figure 6:**
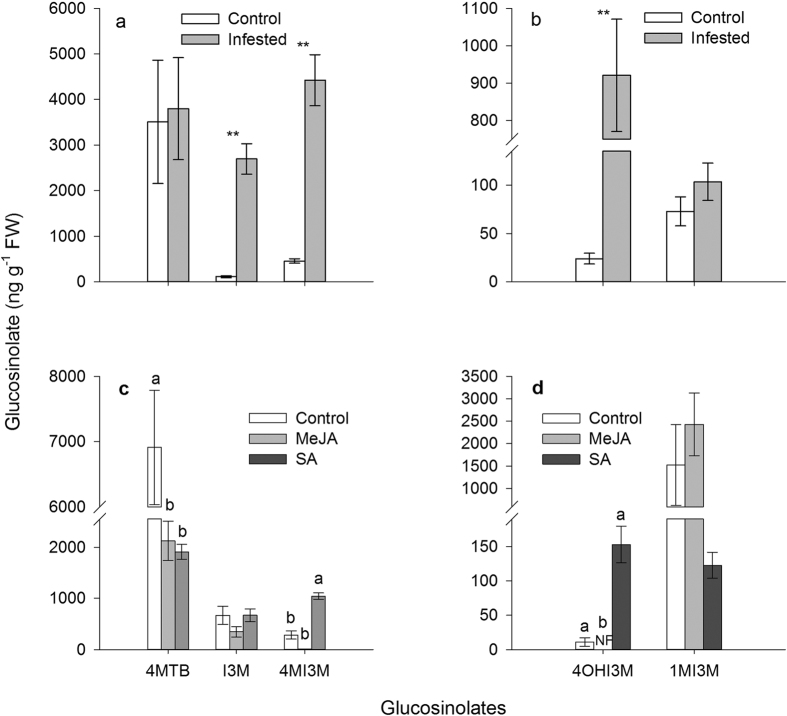
The effects of *M*. *persicae* feeding and methyl jasmonate (MeJA) or salicylic acid (SA) treatment on glucosinolate concentration in plant leaves. (**a,b**) *M. persicae* feeding increased indole glucosinolate concentration in plant leaves. **P < 0.01; Student’s *t*-test. (**c,d**) MeJA and SA treatment on glucosinolate concentration in plant leaves. Kruskal-Wallis test. Different letters above bars indicate significant difference at *P* < 0.05. Values shown are mean ± SE. Glucosinolate side chain abbreviations: 4MTB, 4-methylsulfinylbutyl; I3M, indol-3-ylmethyl; 4MI3M, 4-methoxyindol-3-ylmethyl; 4OHI3M, 4-hydroxyindol-3-ylmethyl; 1MI3M, 1-methoxyindol-3-ylmethyl.

*M. persicae* feeding significantly increased the concentration of indole glucosinolates I3M (3-indolylmethyl) (24-fold increase; *t* = 7.756, df = 6, *P* < 0.001), 4MI3M (4-methoxyindol-3-ylmethyl) (10-fold increase; *t* = 7.049, df = 6, *P* < 0.001), and 4OHI3M (4-hydroxyindol-3-ylmethyl) (38-fold increase; *t* = 5.951, df = 6, *P* = 0.001) in Chinese cabbage leaves (Fig. 6a,b). The indole glucosinolate 4MI3M was also enhanced by SA treatment (3.6-fold increase; Kruskal-Wallis test; *P* < 0.05) (Fig. 6c).

## Discussion

Many insects have been reported to reduce plant resistance by suppressing plant jasmonate signaling, though the underlying mechanism is not clear. For example, the invasive spider mite *Tetranychus evansi* Baker and Pritchard suppressed SA and JA defence signalling pathways in tomato and performed better on previously attacked plants than on control plants^24^. The whitefly, *B*. *tabaci*, activated the *A. thaliana* SA signalling pathway and repressed effectual jasmonate signalling^5^. In addition to suppressing plant defence responses, some phloem-feeding insects can also increase amino acid concentration in host plant. Feeding by the greenbug, *Schizaphis graminum* (Rondani) increased the absolute concentration of amino acids and the proportion of essential amino acids in wheat and barley^25^. In our study, *M*. *persicae* had a faster weight gain and population growth rate when feeding on pre-infested Chinese cabbage plants compared with on non-infested plants, suggesting that *M*. *persicae* can manipulate plant defences or nutrition for their own benefit. In contrast, *M*. *persicae* feeding on *A. thaliana* increased plant resistance and the aphids produced fewer nymphs on pre-infested plants^26^. This could be because the aphids only fed on the plants for 48 h in their study^26^, but in our study the aphids fed on the plants for five days.

Nitrogen is a key factor that limits insect herbivore growth and fecundity, and the nitrogen in plant phloem sap is generally in the form of free amino acids^27^. Thus, the amino acid concentration and composition in plant phloem sap significantly influences aphid performance^20^,^27^. However, phloem sap has a low quality and quantity of amino acid for phloem feeding insects. Some aphids are able to change amino acid composition and concentration in their host plant. For example, *Rhopalosiphum padi* (L.) feeding increased total amino acid concentration in maize and barley^28^. The aphid *Rhopalosiphum insertum* (Walker) feeding on *Sorbus commixta* Hedlund leaves induced gall formation and the aphids developed faster when feeding on the galls that contained more amino acid^29^. In our study, the relative concentrations of the essential amino acids threonine and tryptophan were significantly higher in the phloem sap of the aphid pre-infested Chinese cabbage, suggesting that *M*. *persicae* feeding altered the amino acid composition in plant phloem sap. Also, there was a significant increase in the amino acid:sugar ratio in the phloem sap of the *M*. *persicae* infested plants. The enhanced amino acid:sugar ratio is widely recognized as an indicator of high plant nutritional quality for aphids^27^. Our results imply that the increased performance of *M. persicae* on the pre-infested cabbage plants can partly attribute to higher amino acid:sugar ratio in the phloem sap of infested leaves. The underlying mechanism of how aphids manipulate plant amino acid metabolism remains unknown.

Plant senescence is usually accompanied by increased free amino acid concentration in plant leaves and thus senescent leaves seem to be more susceptible to some phloem-feeding insects^22^,^30^,^31^. *M*. *persicae* showed a shorter pre-reproductive time when feeding on dark-induced senescence *Solanum tuberosum* L. (Solanales: Solanaceae) plants, which possibly had more nutrition in their phloem sap^30^. In our study, feeding by *M*. *persicae* reduced chlorophyll concentration and accelerated leaf yellowing and senescence, which could partly explain the observed increase of amino acid in Chinese cabbage. The expression of SA marker genes in Chinese cabbage increased significantly after *M*. *persicae* feeding and SA signalling pathway has been reported to play a role in controlling leaf senescence, suggesting that SA signaling might involve in *M*. *persicae* induced leaf senescence in this study^32^. In contrast to our results, Pegadaraju *et al.* (2005) found that the *phytoalexin deficient4* (*pad4*) mutant *Arabidopsis*, which had a delayed senescence process in response to *M*. *persicae* feeding, was more susceptible to aphids, while the hypersenescence mutant *Arabidopsis constitutive expresser of PR genes5* and *suppressor of SA insensitivity2* were more resistant to aphids^19^. These results suggest that leaf senescence contributes to plant resistance to aphids^19^. However, later studies found that *PAD4*-dependent resistance was not associated with plant senescence^33^. Therefore, the hypersenescence *Arabidopsis* mutants may not reallocate and recycle nitrogen and thus differ from aphid infested plants that contain higher free amino acid concentration. Moreover, *M. persicae*, like other aphids, may be able to draw plant amino acid from other part of the host plant and thus increased their fitness, whereas hypersenescence mutant Arabidopsis may not be associated with nutrition reallocation^4^,^25^,^29^.

In addition to enhancing plant nutritional quality, *M. persicae* infestation also increased the concentration of defensive glucosinolates. Aphid feeding increased levels of indole glucosinolates I3M, 4OHI3M, and 4MI3M. The glucosinolate 4MI3M is an aphid deterrent indole glucosinolate in *Arabidopsis* and could be induced by *M*. *persicae* feeding^15^. We found that *M*. *persicae* preferred non-infested plant leaves compared with pre-infested leaves, which may be due to high 4MI3M concentration in pre-infested leaves. However, increase of glucosinolate concentration in *M*. *persicae* feeding or SA-treated plant leaves did not reduce aphid performance, which could be explained by the following two reasons. First, myrosinase is produced in cells adjacent to the phloem and aphids could ingest phloem sap without contacting myrosinase and thus avoid breaking down glucosinolates to more toxic products^34^. Alternatively, the negative impacts of glucosinolates in pre-infested plants on aphid performance may be masked by increased nutrition in plant phloem sap.

The role of jasmonate and SA in plant resistance to aphids is not consistent among plant species^35^,^36^. In our study, the expression levels of SA related genes were significantly enhanced by aphid feeding, while jasmonate marker genes were less affected, which was consistent with previous studies on aphid-Arabidopsis interaction^37^,^38^. The resistant tomato accumulated stronger and faster SA marker genes in response to *M*. *persicae* feeding, while aphid growth on SA-signalling mutant, *nonexpresser of PR genes1*, was comparable with that on wild-type plant^19^,^39^. Application of MeJA or SA on wheat had no significant effects on *Sitobion avenae* (Fabricius) performance^40^. Nymphs of the pea aphid *Acyrthosiphon pisum* Harris feeding on pre-infested broad bean *Vicia faba* L. took a shorter time to reproduce than on non-infested plants^41^. Although *A*. *pisum* feeding reduced JA concentration in *V*. *faba*, JA treatment did not significantly influence aphid development time, implying that factors other than jasmonate signalling are responsible for the enhanced performance of *A*. *pisum* feeding on pre-infested *V*. *faba*^41^. In our study, *M*. *persicae* performance was comparable on MeJA-treated, SA-treated, and control plants, suggesting that exogenous treatment of cabbage plants with these chemicals does not affect aphid performance. However, jasmonate signalling plays a significant role in inducing plant volatiles, which serve as cues for natural enemies to locate pests. Further study is needed to examine the effects of *M*. *persicae* feeding on volatile production of Chinese cabbage as well as the searching behavior of the aphid’s natural enemies.

**Figure 7:**
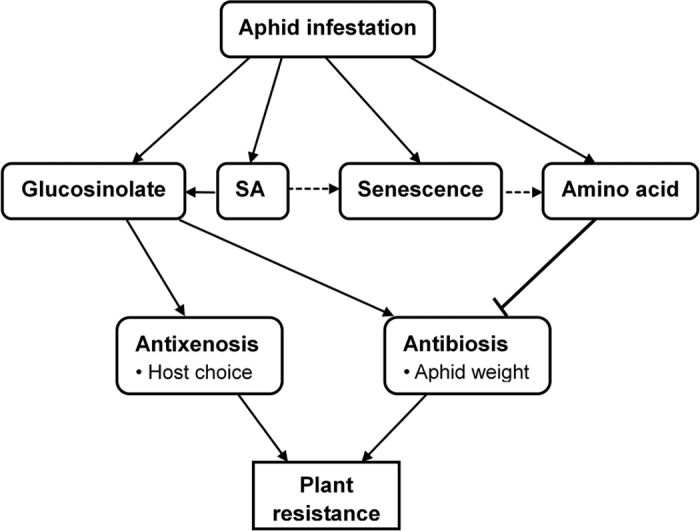
Model of the interaction between *M. persicae* and Chinese cabbage plants. *M. persicae* infestation activated salicylic acid (SA) signaling pathway and increase accumulation of glucosinolates in Chinese cabbage leaves, which have antibiotic and antixenotic effects to *M. persicae*. SA is a positive regulator of glucosinolates and SA possibly promote plant senescence that may contribute to the increase of free amino acid in plant leaves^23^,^32^. Amino acid is a key nutrition for aphids^20^,^21^. Thus, *M persicae* reduce direct resistance of Chinese cabbage likely contributes to the higher nutritional quality in infested leaves. Solid arrows indicate promotion or positive modulation of the processes and dashed arrows indicate possible promotion of the processes. Vertical bar indicates negative modulation of the process.

In summary, our results suggest that *M. persicae* could enhance plant nutritional quality for their own benefit possibly by accelerating leaf senescence process. Although *M. persicae* feeding also enhanced glucosinolate concentration, the negative impacts of glucosinolates may be compromised by the aphid detoxification system or masked by the nutritional quality increase in plant phloem sap. According our results, we propose a model depicting *M. persicae*-Chinese cabbage interaction (Fig. 7). These findings suggest that both toxic compounds and nutritional quality in host plant should be considered in the research of insect-plant interaction. Whether aphid infestation accelerates plant senescence and increases amino acid concentration in plant phloem sap is a common phenomenon and the underlying mechanism need further investigation.

## Methods

### Plants and insects

Seeds of Chinese cabbage (c.v. ‘QingZa 3’) were germinated in 9 cm Petri dishes for 24 h at room temperature and then were grown in 250 mL pots containing soil mixture (peat moss:perlite = 4:1) in a walk-in growth chamber (24 ± 1 °C, 60% RH, 16:8 L/D). Plants were watered with tap water as necessary. Unless otherwise stated, two-week-old plants were used for all experiments. *M. persicae* were reared on cabbage (*Brassica oleracea*; c.v. ‘Qingan 70’) in a greenhouse.

### Aphid performance assays

Twelve apterous *M*. *persicae* adults were confined on the first leaf of the Chinese cabbage plant using a nylon mesh bag. Control plants were caged by empty nylon mesh bags. Cotton was wrapped around the petioles to prevent mechanical damage to the plants. After feeding for five days, all aphids were removed using a hairbrush, and then 2–3 apterous adult aphids were introduced to each of the first leaf. After another 24 h, the adults were removed, leaving 5–8 nymphs on each leaf. The nymphs on the leaves were collected and weighed on a microbalance (resolution 0.001 mg; Sartorius MSA 3.6 P-000-DM, Gottingen, Germany) after indicated days. This experiment repeated for four times, which lasted for 3–7 days. The mean weight of the aphids on each plant was considered as one replicate and eight replicates were performed for this assay. To evaluate the influence of plant defence signals, jasmonate and SA, on aphid performance, we sprayed 1 mM MeJA, 1 mM SA or carrier solution (MilliQ water containing 0.05% Tween 20) on the Chinese cabbage plants, and then examined aphid performance similarly (eight replicates for each treatment). We also assessed the influence of aphid feeding on population growth of *M*. *persicae*. Five one-day-old nymphs were introduced to each pre-infested leaf (feeding for four days by 20 third instar nymphs to adults) or to a control leaf, and the total number of aphids on each plant was counted after another seven days (10–12 replicates for each treatment). The introduced aphids were then weighed as described above.

### Host preference of *M. persicae*

The first leaf of each Chinese cabbage plant was infested with 12 apterous *M*. *persicae* adults, or caged with an empty nylon mesh bag as described above. After five days, one infested and one control leaves were put in a Petri dish (9 cm diameter). Leaf petioles were wraped in cotton and the leaves were inserted in Petri dishes through two holes drilled along the diameter and thus the leaves can be kept intact. Then, about 18 alate *M. persicae* adults were introduced to the center of the Petri dish. After 2 and 8 h, the number of adult aphids on each leaf was counted. Eleven replicates were conducted for this assay.

### Chlorophyll analysis

Chlorophyll content is a reliable marker of plant senescence, which associated increase of free amino acid in plant leaves. The first leaf of each Chinese cabbage plant was infested with 12 *M*. *persicae* adults or caged with an empty cage. After 5 days, the leaves were collected for chlorophyll analysis. Total chlorophyll in the plant leaves was extracted by ethanol and determined as described previously^42^. Eight replicates were performed for this assay.

### Amino acid and sugar analysis

To investigate whether *M*. *persicae* can manipulate plant nutritional quality, we measured amino acid, sucrose, fructose, and glucose in the phloem sap and leaves of Chinese cabbage plants. Because the abundance of trehalose, lactose, and melezitose are less than 1 ppm in Chinese plants, we did not consider these sugars in this study. All of the reagents used in this study were purchased from Sigma-Aldrich (St. Louis, MO, USA). The plant samples were collected after 12 aphids feeding for five days. Amino acid and sugar in leaves were extracted with 0.1 M HCl by grinding with a glass mortar and pestle (eight replicates for each treatment)^43^, while amino acid and sugar in the phloem sap were collected by immersing the petiole of the leaves in 600 μL 5 mM EDTA (pH = 7.0) solution for 4 h in a dark growth chamber (25 °C, 100% RH) (eight replicates for each treatment)^44^. EDTA effectively enhanced the exudation of phloem sap from cut petioles, while it caused little contamination from xylem or cut cells^44^. Amino acids were analysed as described previously^43^. The sugars (sucrose, glucose, fructose) extracted from plants were analysed by a LTQ XL linear ion trap mass spectrometer (Thermo Scientific, Waltham, MA, USA). Liquid chromatography separations were carried out with XBridge Amide Column (100 mm × 2.1 mm; Waters Corp., Milford, MA, USA). Sugar elution was performed by applying isocratic elution (mobile phase was 85/15 acetonitrile/water with 0.1% ammonium hydroxide) for 40 min. The flow rate was 0.2 mL/min. The mass spectrometer worked in the negative electrospray ionization (ESI) mode. Nitrogen was used as the sheath gas (30.0 arbitrary units) and auxiliary gas (5.0 arbitrary units). The spray voltage was set at 4.5 kV and the ion transfer capillary temperature was 275 °C. The sugars were scanned and fragmented using data dependent MS/MS. Masses of precursor and product ions and collision energy for each sugar were as described in the supplementary Table S1. Data were acquired and processed using Xcalibur 2.1 software (Thermo Scientific, Waltham, MA, USA). Quantification was achieved by external standard sugars mixture of known concentrations.

### Glucosinolate analysis

Twelve apterous aphid adults were confined on the first leaf of a Chinese cabbage plant as described and control plants were individually caged by empty nylon mesh bags. After the aphids had fed for five days, the leaves were collected, weighed and stored at −80 °C until analysis (seven replicates for each treatment). To determine defence signals on glucosinolate concentration of the Chinese cabbage plants, we treated the plants with MeJA, SA or carrier solution as described above, and collected the leaves three days after treatment (seven replicates for each treatment). Glucosinolates were extracted and analysed as described previously^45^. Briefly, 100 mg leaves were put in a 1.5 mL centrifuge tube and kept in 96 °C hot water for 3 min to inactivate the mycrosinase. The leaves were then ground with glass mortar and pestle, and then 1000 μL MilliQ water was added. The mixture was then centrifuged at 12,000 g, 4 °C for 15 min. The supernatant was collected and passed through 0.22 μm syringe filters before analysis. Glucosinolates were analysed as described previously^45^. The glucosinolate amounts were calculated according to a standard curve made by 2-propenyl glucosinolate (sinigrin).

### RNA extraction and analysis

To investigate the influence of *M*. *persicae* feeding on Chinese cabbage defence signalling pathways, we analysed the jasmonate and SA marker genes changes in response to aphid feeding. The *β-1*, *3-GLUCANASE 2* (*BrBGL2*) and *PATHOGENESIS-RELATED 1* (*BrPR1*) are marker genes for the SA pathway, while the *LIPOXYGENASE 2* (*BrLOX2*) and *VEGETATIVE STORAGE PROTEIN 2* (*BrVSP2*) are marker genes for the JA pathway. The *ACTIN 2* (*BrACT 2*) gene was used for normalization. The primer sequences and GenBank accession numbers used for quantitative RT-PCR can be found in supplementary Table S2^46^. The first leaf of each Chinese cabbage plant was infested with 12 *M. persicae* adults or caged with an empty cage. After one, three, and five days, the leaves were detached, frozen in liquid nitrogen, and stored at −80 °C. To investigate the effects of MeJA and SA on expression of these marker genes, we sprayed 1 mM MeJA, 1 mM SA or carrier solution (MilliQ water containing 0.05% Tween 20) on the Chinese cabbage plants, and collected samples two days after treatment. Total RNA (1 μg) was isolated with the RNAiso Plus (Takara Biotechnology CO., LTD). First-strand cDNA was synthesized using the PrimeScript RT Reagent Kit (with gDNA eraser; Takara). Real-time quantitative RT-PCR was performed on an iQ5 Multicolor Real-Time PCR Detection System (Bio-Rad Laboratories, Hercules, CA, USA) with SYBR Premix Ex Taq II (Tli RNaseH Plus; Takara). The PCR was performed under the following conditions: 95 °C for 3 min, followed by 40 cycles of 95 °C for 5 s, 58 °C for 30 s. The relative expression of marker genes and the statistical analysis of relative expression results were analysed by the REST-2009 Software (Qiagen, Hilden, Germany) using the Ct values^47^.

### Statistical analysis

Statistical analysis was conducted using the IBM SPSS Statistics package (version 19.0; SPSS Inc., Chicago, IL, USA). Aphid weights on control and infested plants, amino acid concentration, sugar concentration and glucosinolate concentration in control and infested plants were analysed using Student’s *t*-test. The numbers of nymphs produced per nymph after feeding on plants for seven days were compared by nonparametric test Mann-Whitney *U* test. Numbers of *M*. *persicae* settled on control and pre-infested plants were compared using Chi-square test to test the null hypothesis that the proportion of responding aphids (%) does not differ between pre-infested and control plants. Aphid weight and glucosinolate concentration on control, MeJA, and SA-treated plants were analysed with the Levene’s and Kolmogorov-Smirnov tests to determine homogeneity of the variances and normality and then the data were analysed using one-way analysis of variance (ANOVA); and means were compared by Tukey’s HSD (honest significant difference) test at *P* < 0.05. The data that did not pass these two tests were analysed using the nonparametric Kruskal-Wallis test.

## Additional Information

**How to cite this article**: Cao, H.-H. *et al.* The green peach aphid *Myzus persicae* perform better on pre-infested Chinese cabbage *Brassica pekinensis* by enhancing host plant nutritional quality. *Sci. Rep.* **6**, 21954; doi: 10.1038/srep21954 (2016).

## Supplementary Material

Supplementary Information
