# Corrigendum: The green peach aphid *Myzus persicae* perform better on pre-infested Chinese cabbage *Brassica pekinensis* by enhancing host plant nutritional quality

**DOI:** 10.1038/srep43076

**Published:** 2017-03-09

**Authors:** He-He Cao, Hui-Ru Liu, Zhan-Feng Zhang, Tong-Xian Liu

Scientific Reports 6: Article number: 2195410.1038/srep21954; published online: 02 24 2016; updated: 03 09 2017

In the original version of this Article, Affiliations 1 and 2 were incomplete. The correct affiliations are listed below.

State Key Laboratory for Crop Stress Biology in Arid Areas, Northwest A&F University, Yangling, Shaanxi, 712100, China.

Key Laboratory of Crop Pest Management on the Northwest Loess Plateau, Ministry of Agriculture, Northwest A&F University, Yangling, Shaanxi, 712100, China.

These errors have been corrected in both the HTML and PDF versions of the Article.

