# Supplementary material for: The green peach aphid Myzus persicae perform better on pre-infested Chinese cabbage Brassica pekinensis by enhancing host plant nutritional quality
Source: Sci Rep. 2016 Feb 24;6:21954. doi: 10.1038/srep21954 (PMC4764936; doi:10.1038/srep21954)
Supplement: Supplementary Information [file srep21954-s1.pdf]

# Supplementary Information

## The green peach aphid *Myzus persicae* perform better on pre-infested Chinese cabbage *Brassica pekinensis* by enhancing host plant nutritional quality

He-He Cao[1,2], Hui-Ru Liu[3], Zhan-Feng Zhang[1,2] and Tong-Xian Liu[1,2*]

[1]State Key Laboratory for Crop Stress Biology for Arid Areas, and [2] Key Laboratory of Crop Pest Management on the Northwest Loess Plateau, Ministry of Agriculture, and [3] Innovation Experimental College, Northwest A&F University, Yangling, Shaanxi, 712100, China

**Correspondence should be addressed to Tong-Xian Liu (txliu@nwsuaf.edu.cn); Mob. +86-029-87092663**

**Figure S1 | *M. persicae* performance on non-infested control (Control) and previously infested (Pre-infested) plants.** Aphid weight after feeding for three days (a), four days (b), and seven days (c) on control and pre-infested plants. This experiment repeated with different sets of aphids and plants. Values shown are mean ± SE. **$P < 0.01$; Student's *t*-test.

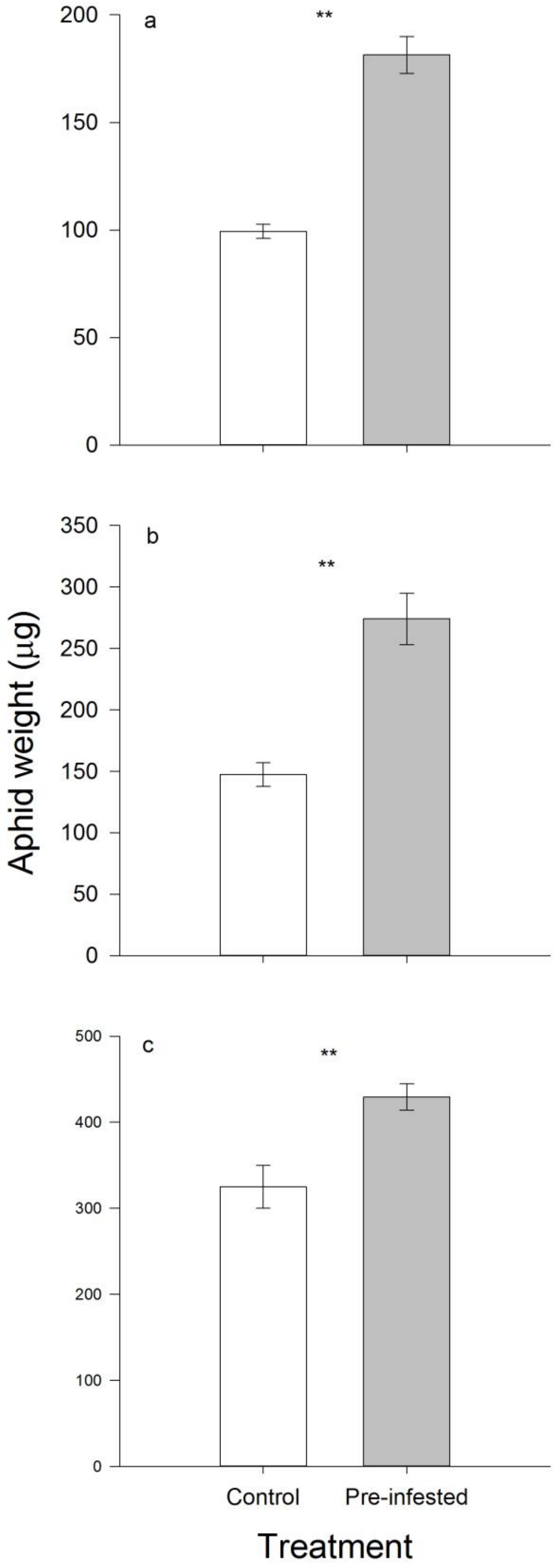

**Figure S2 | Chlorophyll concentration in plant leaves.** Chlorophyll concentration in control plant leaves or leaves infested with aphids for five days. $^{**}P < 0.01$; Student's $t$-test. Values shown are mean ± SE.

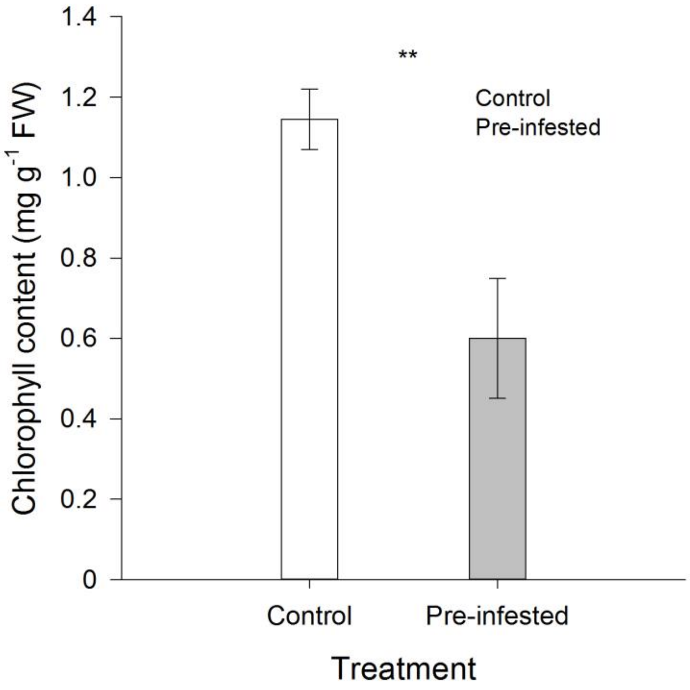


**Table S1 | Masses of precursor and product ions and collision energy for sugars.**

| Sugar | Precursor ion [M-H]-(*m/z*) | Product ion (*m/z*) | Collision energy (eV) |
|---|---|---|---|
| Fructose | 179 | 143 | 35 |
| Glucose | 179 | 143 | 35 |
| Sucrose | 341 | 179 | 35 |

**Table S2 | Nucleotide sequences and GenBank accession numbers for primers.**

Nucleotide sequences for the Chinese cabbage *Brassica pekinensis* gene-specific primers in this study were as follows:

| Gene | Forward primer | Reverse primer |
|---|---|---|
| *BrVSP2* | 5'-GACTCCAAAACGGTGTGCAAA-3' | 5'-AGGGTCTCGTCAAGGTCAAAGA-3' |
| *BrLOX2* | 5'-TCCCCACTTCCGCTACACC-3' | 5'-AATACTTTCCGGGCCAGAAAC-3' |
| *BrBGL2* | 5'-GCAGAACATCGATAGAGCGGT-3' | 5'-TGAATGTCCCACTCGAAGGC-3' |
| *BrPR1* | 5'-TACGCTCAAAACTACGCCGA-3' | 5'-GAAAGGTCCCCGCTACTTCC-3' |
| *BrACT2* | 5'-ACCCAAAGGCCAACAGAGAG-3' | 5'-CTGGCGTAAAGGGAGAGAACA-3' |

The GenBank accession numbers for these genes are as follows:*BrPR1*(BBRAF03K11), *BrBGL2* (BBRAF10P08), *BrVSP2*(EX103556), *BrLOX2* (EX100417), and *BrACT2*(BBRAF03F20).

These primers sequences and GenBank accession numbers were cited from: Abe, H. et al. Development of full-length cDNAs from Chinese cabbage (*Brassica rapa* subsp. *pekinensis*) and identification of marker genes for defence response. *DNA Res*. **18**, 277-289 (2011).
